# Use of dementia and caregiving-related internet resources by informal caregivers: A cross-sectional study

**DOI:** 10.3389/fmed.2022.978635

**Published:** 2022-09-14

**Authors:** Soraia Teles, Constança Paúl, Cristina Costa-Santos, Ana Ferreira

**Affiliations:** ^1^Department of Behavioural Sciences, School of Medicine and Biomedical Sciences, University of Porto (ICBAS-UP), Porto, Portugal; ^2^Center for Health Technology and Services Research (CINTESIS), Porto, Portugal; ^3^Faculty of Medicine, University of Porto (FMUP), Porto, Portugal

**Keywords:** dementia, caregiving-informal, information technology, health behavior (MeSH), internet

## Abstract

Informal dementia caregivers are at greater risk of experiencing physical and mental health issues as compared to the general population. Internet-based resources may provide accessible opportunities to backing informal dementia caregivers by addressing their information and support needs. This cross-sectional study aims to characterize the use of dementia and caregiving-related internet resources by caregivers and identify variables associated with such use. Primary data were collected through a web-based survey (*N* = 158). Linear regression models were used to assess the associations of predisposing, enabling, and need variables with the frequency of using the internet for caregiving-related purposes. Most caregivers (93%) have ever used the internet to gather general information about dementia. The frequency of using internet resources was, however, moderate. The multivariable linear regression model suggests that being younger (β = −0.110, *p* = 0.009), not having a source of support to provide care (β = −2.554, *p* = 0.012), having used a face-to-face psychosocial intervention at some point (β = 2.731, *p* = 0.003), being employed (β = 2.558, *p* = 0.013), and appraising one's own physical health negatively (vs. appraising it as similar; β = 3.591, *p* < 0.001), are associated with a higher frequency of using caregiving-related internet resources. Our findings confirmed the association of age and perceived health status with caregiving-related internet use reported in other studies. The role of enabling variables as lifetime access to psychosocial interventions and having a usual source of support to provide care was a new addition. This study informs the design and deployment of information and support to dementia caregivers.

## Introduction

Fifty million people are estimated to live with dementia around the globe (1). This group of disorders is a main cause of dependence among older adults (2). Worldwide, families are the backbone of long-term care provided to people with dementia (PwD) and carry much of the burden of the disease (3).

Throughout the caregiving trajectory, informal dementia caregivers are at greater risk of experiencing depression and anxiety disorders, when compared both to the general population and to caregivers of people living with other chronic diseases (4). Informal caregivers tend to report poor diet, unhealthy sleep and poor exercise habits and often face financial strain, isolation, and discrimination (5, 6).

Higher levels of stress are thought to be experienced by caregivers who perceive their coping resources as being beneath their demands (7). To deal with caregiving, caregivers may rely on a plethora of information and support sources, including care professionals, community services, printed information materials, and internet-based resources (8).

The increasing attention given to internet interventions aimed at dementia caregivers has put in perspective the role that web resources may play in the upcoming years. Such resources may diverge in complexity, ranging from static informational web platforms to interactive and customizable web programs/apps providing psychoeducation, training, or health support (9). Internet-based resources may provide accessible and affordable opportunities to tackle barriers faced by caregivers in using in-person psychosocial services. Geographic inequalities concerning service offers, 24/7 or very intensive care, transportation issues, or experiencing stigma, are among the reasons hampering the access of caregivers to in-person support (2). Dementia caregivers have shown interest in using internet resources to find support, connect with other caregivers, and seek information about dementia, financial, emotional, and practical aspects of caregiving (10–12).

Only a few studies have explored the factors associated with the use of internet-based health and caregiving-related resources by dementia caregivers (5, 11–20). Most studies resort to heterogeneous samples of caregivers (caring in different chronic diseases), and findings were inconsistent on the factors associated with the use of health and caregiving-related internet resources. The use of such resources was associated in some studies (but not in others) with caregivers' age (5, 13, 15), education (5, 13, 15), income (15), hours spent caring (15), relationship with the care recipient (15), emotional stress (15, 16), and perceived health status (5). Qualitative research has stressed the role of burden of care (20), stage of the disease (14), years spent caring, social support, and self-efficacy (17). Conflicting literature findings may result, among other reasons, from a substantial heterogeneity in the inquiry process that supports the categorization of individuals as users or non-users of internet resources. Most studies base such categorization on generic questions about health or caregiving-related internet use, with no further specification of such purposes. Much less provide information on the use of the internet for specific purposes (13). Online health, dementia, or caregiving-related resources are highly diverse regarding the information they convey and pose different user interaction demands (passive as, e.g., in an informational website, vs. active, e.g., in a psychoeducational program). The motivations and the profiles of caregivers may differ among those searching for information, looking for training or support on practical care, or searching for resources on self-care. A disaggregated analysis of how different factors may relate to different internet uses is missing in the literature. Moreover, caregivers are usually categorized according to the dichotomy of being users or non-users of the internet for health, dementia, or caregiving-related purposes, a grouping that presents many limitations in explaining caregivers' resource seeking behavior (15). In a digital era, non-users of the internet, both in general and for health-specific purposes (at a very minimum, basic online searches), are becoming less prevalent in developed countries. At least for research conducted in those countries, non-users of health-related internet resources are becoming circumscribed to the most economically, socially, and digitally excluded groups in society. Research comparing users and non-users of health resources on the web is at risk of confusing factors associated with such use, with those related to digital literacy. A more fine-grained analysis is required, focusing on individuals who can use the internet, as well as on the factors associated with the frequency of using—rather than the use/non-use- of dementia and caregiving-related internet resources.

Another caveat of studies in this field is the scarce attention given to “enabling” or “need” variables (21) such as caregivers' service utilization history or physical/psychological health, contrasting to the great focus on predisposing variables (15).

In tackling current research gaps, this cross-sectional study investigates the information and support-seeking behavior of dementia caregivers focusing on the utilization of internet resources. Our objective is threefold: 1) describe the use of dementia and caregiving-related internet resources in a sample of informal dementia caregivers who can use the internet autonomously, regarding the frequency and purposes of use; 2) compare dementia caregivers who characterize differently on predisposing, enabling, and need variables on their frequency of using dementia and caregiving-related internet resources (for eight different purposes); and 3) identify predisposing, enabling, and need variables which are associated with the overall frequency (composite measure) of using dementia and caregiving-related internet resources. The study was conducted in Portugal, a country with a high prevalence of dementia (21 cases per 1,000 inhabitants) (22), and high rates of informal co-residential care (23).

## Materials and methods

### Type of study

Cross-sectional study design resorting to quantitative data collection techniques (e-survey).

### Participants

Primary data were collected from a non-probabilistic sample of informal dementia caregivers recruited nationally (Portugal). Participants were recruited through advertising on 1) the online platforms of the National Alzheimer's Association; 2) social media groups on caregiving issues; and 3) the online channels of community-based projects, identified from a comprehensive national list (24). Eligibility criteria were appraised through trial questions opening the e-survey and include: 1) being aged 18 or older; 2) providing unpaid care; to 3) a person with dementia (PwD); 4) living in the community. Complete information about the study was disclosed on the introductory page of the e-survey and consent for participation was obtained. This study was approved by the Ethics Committee for Health of the São João University Hospital Center/Faculty of Medicine of the University of Porto (reference 208/18).

### Variables and measures

An e-survey was designed to be self-completed by participants. The survey was disseminated as part of a larger project on the assessment of support needs of dementia caregivers. Lime Survey was used to prepare the questionnaire in a fill-in form. That includes information on predisposing, enabling, and need variables about both the caregiver and the care recipient. Variables were classified as predisposing, enabling or need variables according to the behavioral model proposed by Andresen and Aday (21)—as one of the most used frameworks to examine health or social services use—regarding the characteristics of the population at risk/service user. Data on the use of dementia and caregiving-related internet resources were also collected. Eight specific purposes of internet use were addressed, which were derived from a narrative literature review performed by the authors to develop the e-survey. The questions on the use of internet resources consisted of a subjective measure of frequency (ranging from “never” to “frequently”) rather than on a self-report measure of hours spent online *per* purpose. Previous research found low levels of accuracy for self-report measures of time spent online, more especially among persons with high levels of internet usage (25). See Table 1 for the variables covered for this study.

**Table 1:** e-Survey: variables covered and retrieved for this study.

**Variable category**	**Variables**
Predisposing—carer	Age; gender; years of schooling; marital status; relationship with the person with dementia; cohabitation with the person with dementia (yes/no)
Predisposing—care recipient	Age; gender
Enabling	Carer employment status (employed/not employed); having a usual source of support to assist the carers in their caregiving role caregiving (yes/no); Lifetime access to any psychosocial intervention (having accessed at least once in a lifetime to any of the following interventions: psychoeducational programs; support groups; mutual-aid groups; individual psychological support; specialized counseling on dementia—dichotomized into yes/no)
Need	Number of years caring; number of hours caring (per week); type of dementia (Alzheimer's, Vascular, Frontotemporal; Lewy body; other, unknown); care recipient perceived level of dependence (mild/moderate/severe/total); self-perception (carer) of physical health; and self-perception (carer) of mental health (rated as much worse/worse; similar; better/much better than counterparts of the same age and gender).
Dementia and caregiving-related internet use	Never, rarely, sometimes or frequently have used the internet for: 1. gathering generic information on dementia disorders, including causes, types of dementia, symptoms, progression, diagnosis, and pharmacological/non-pharmacological interventions; 2. gathering information to learn about strategies to provide good quality care; 3. finding professional care services for the person with dementia; 4. finding support services aimed at supporting the caregiver, including, for instance, counseling, support groups and other interventions; 5. learning about strategies to manage negative psychological consequences of providing care, for instance, anxiety and depression symptoms; 6. searching information on legal, fiscal and financial support topics and measures; 7. reading about and/or sharing experiences with other dementia carers on social media (e.g., Facebook pages, blogs); and 8. Benefiting from internet interventions targeted at informal dementia carers.

All data collected, including care recipient information, were based on the caregivers' subjective assessment. The survey was open for 6 months (second and third quarters of 2019) and data were hosted in the secure servers of the University of Porto.

### Data analysis

The first study goal was addressed by computing descriptive statistics to characterize the participants and their use of dementia and caregiving-related internet resources. To address the second study goal, the Spearman's Rho Test was used to study the associations of frequency of internet use (for each of eight different dementia and caregiving-related purposes) with continuous or ordinal variables, while either the Mann-Whitney *U* Test or the Kruskal Wallis H Test were used to compare two or more groups.

To address the third study goal, we first examined the factorial structure of questions on the frequency of using dementia and caregiving-related internet resources (8 items). A Principal Component Analysis based on polychoric correlations was conducted utilizing the FACTOR software (26). The sample size (*N* = 158) sufficed to perform this analysis with eight items, complying with the minimum ratio of seven participants per variable [COSMIN guidelines; (27)]. The inter-item correlation matrix, the Bartlett test of sphericity (28), and the KMO test (29) were used to evaluate the adequacy of the data to perform the analysis. The recommended KMO test value is of at least 0.6 (29) while the Bartlett's test of sphericity tests the null hypothesis that there is no relationship between the items. To decide on the number of factors to retain, we considered both the Kaiser's criterion retaining factors with eigenvalues >1, and the Parallel Analysis (30) comparing the size of the eigenvalues with those from a randomly generated data set. Accounting for the unifactorial solution derived from the factor analysis (see Results), an unweighted sum of all eight items was computed. This composite variable was used as the dependent variable in univariable and multivariable linear regression models (see Results).

In selecting the candidate independent variables for the multivariable model, simple linear regressions were performed with each predisposing, enabling and need variables in Table 1. Categorical variables with more than two categories were dummy coded. Variables theoretically relevant for the model, or variables in which a *p* ≤ 0.25 was identified in the univariable analyses, were included in the multivariable regression modeling (backward method). Smaller *p* levels as 0.05 can fail in identifying important variables to the overall model (31). Preliminary analyses were conducted to ensure non-violation of the assumptions of normality, independence of residuals, linearity, multicollinearity, and homoscedasticity. Variables with a significant contribution to the dependent variable (*p* < 0.05) or those theoretically relevant were kept into the final model. The sample size (*N* = 158) would suffice to conduct the multiple regression with a good number of independent variables, accounting for the general reference of 10 cases per candidate variable. All *p*-values are two-sided, and the statistical significance was considered for *p* < 0.05. Data analysis was performed with SPSS 26.

## Results

### Participants

The e-survey was accessed by 308 potential participants. Upon the visualization of the study information page, 14.9% (*n* = 46) left the survey, and 5.5% (*n* = 17) were stopped from advancing after declaring to be paid caregivers. From the remaining 245 participants, 83.3% (*n* = 204) filled in sociodemographic information. Nine cases were removed due to noncompliance with inclusion criteria (caring for someone with a condition other than dementia). Thirty-seven participants left the survey before answering the questions on the usage of caregiving-related internet resources. This withdrawal resulted in a final sample of 158 participants. In considering the 236 eligible cases, we obtained a dropout of 33.1%, which is in line with the average dropout (30%) for online surveys (32).

Most participants were daily internet users (91.1%, *n* = 143), and the remaining reported using the internet at least once a week. Table 2 describes the sample. Most caregivers self-identify as female (94.9%), the participants' mean age is 50.2 years, and most are highly schooled (M 14.5 years). Concerning the subjective assessment of their health compared to counterparts of the same age and gender, 49.4% of caregivers consider their physical health worse, and 59.3% believe to be in worse mental health. Most caregivers are children or grandchildren of the PwD (80.4%) and live with the care recipient (70.1%). Participants tended to provide long-term (M 5.7 years) and intensive care (Mdn 38 h. per week), even though many have a usual source of support to assist them in their caregiving role (64.5%).

**Table 2:** Sample characterization on predisposing, enabling, and need variables (*N* = 158).

**Predisposing variables—carer**	**All carers**
Age (years), mean (SD)	50.2 (11.8)
Female gender, *n* (%)	150 (94.9)
Years of schooling, mean (SD)	14.5 (4.3)
Marital status, partnered, *n* (%)	85 (53.8)
Relationship with the person with dementia	
Offspring, *n* (%)	127 (80.4)
Spouses, *n* (%)	24 (15.2)
Other, *n* (%)	7 (4.4)
Cohabitation, yes, *n* (%)	108 (70.1)
**Predisposing variables—care recipient**	
Age (years), mean (SD)	78.2 (9.4)
Female gender, *n* (%)	113 (71.5)
**Enabling variables**	
Carer employment status, employed, *n* (%)	99 (63.9)
Support for caregiving, yes, *n* (%)	100 (64.5)
Lifetime access to psychosocial intervention, yes, *n* (%)	77 (49)
**Need variables**	
Type of dementia	
Alzheimer's disease, *n* (%)	82 (52.6)
Vascular dementia, *n* (%)	34 (21.8)
Other, *n* (%)	23 (14.7)
Unknown, *n* (%)	17 (10.9)
Care recipient dependence level	
Mild/moderate, *n* (%)	53 (33.8)
Total/severe, *n* (%)	104 (66.2)
Hours caring (per week), median (IQR)	38 (73.5)
Caregiving duration (years), mean (SD)	5.7 (3.9)
Carer perceived physical health	
Much worse or worse, *n* (%)	77 (49.4)
Similar, *n* (%)	55 (35.3)
Better or much better, *n* (%)	24 (15.3)
**Carer perceived mental health**	
Much worse or worse, *n* (%)	93 (59.3)
Similar, *n* (%)	38 (24.2)
Better or much better, *n* (%)	26 (16.5)

n, number of participants; SD, standard deviation; IQR, interquartile range.

Most care recipients are female (71.5%) and are 78.2 years old on average. The most frequent diagnosis is of Alzheimer's disease (52.6%). According to caregivers' assessment, most care recipients (66.2%) are severely or totally dependent on others to perform their daily activities.

### Use of dementia and caregiving-related internet resources

The frequency of internet use for different dementia and caregiving-related purposes is described in Table 3. Caregivers reported using the internet more frequently to gather generic information on dementia disorders (46.2% used frequently, Mdn 3). For all other purposes, the use of internet resources was mostly of moderate frequency. Lower internet usage was reported for sharing experiences with other caregivers online (Mdn 2), and most caregivers have never used internet interventions (51%, Mdn 1). Overall, there is less frequent use of the internet by caregivers to find support for themselves than to learn more about the disease and support the PwD.

**Table 3:** The reported frequency of internet use for dementia and caregiving-related purposes: descriptive statistics (*N* = 158).

**Variables**	**Never *n* (%)**	**Rarely *n* (%)**	**Sometimes *n* (%)**	**Frequently *n* (%)**	**Mdn (IQR)**
Gather information on disorders	11 (7)	9 (5.7)	65 (41.1)	73 (46.2)	3 (1)
Learn strategies to provide care	13 (8.4)	10 (6.5)	71 (45.8)	61 (39.4)	3 (1)
Find professional care services	16 (10.2)	28 (17.8)	64 (40.8)	49 (31.2)	3 (2)
Manage psychological effects from caring	36 (22.9)	38 (24.2)	44 (28)	39 (24.8)	3 (1.5)
Find support for carers/themselves	25 (15.9)	38 (24.2)	57 (36.3)	37 (23.6)	3 (1)
Find legal/financial information	37 (23.7)	41 (26.3)	43 (27.6)	35 (22.4)	2.5 (1)
Share experiences with other carers	71 (45.2)	33 (21)	34 (21.7)	19 (12.1)	2 (2)
Use internet interventions	80 (51)	42 (26.8)	25 (15.9)	10 (6.4)	1 (1)

n, number of participants; IQR, interquartile range.

### Associations with predisposing, enabling, and need variables

To discern differential associations of predisposing, enabling, and need variables with using the internet for specific dementia and caregiving-related purposes, the analyses were run separately for each of the eight purposes under study. The results are displayed on the Supplementary Table.

Regarding predisposing variables, i) caregivers' age and ii) relationship with the PwD were significantly associated with the frequency of internet use for more than one caregiving-related purpose. Significant negative correlations were found between caregivers' age and the frequency of using the internet to learn about strategies to provide quality care (*r*_s_ = −0.167, *p* = 0.037), and to search for legal, fiscal, and financial information (*r*_s_ = −0.170, *p* = 0.034), with younger caregivers using the internet more for these purposes. Offspring and spousal caregivers differed in their frequency of internet use to gather generic information on dementia (*U* = 1101.5, *z* = −2.358; *p* = 0.018) and to learn strategies to provide quality care (*U* = 1011.5, *z* = −2.454; *p* = 0.014), with the former using the web more frequently for such purposes.

Concerning the enabling variables, i) caregivers' employment status; ii) having a usual source of support to provide care; and iii) lifetime access to any psychosocial intervention, yielded results of statistical significance. The frequency of internet use to learn how to provide quality care (*U* = 2158.5, *z* = −2.124; *p* = 0.034), and to find professional care services for the PwD (*U* = 1979, *z* = −2.964; *p* = 0.003), was higher for employed caregivers. Caregivers not having a usual source of support to provide care report a more frequent use of the internet to share experiences with other caregivers (*U* = 2183.5, *z* = −2.160; *p* = 0.031). Caregivers who have ever benefited from a psychosocial intervention used the internet more frequently to learn strategies to manage negative psychological consequences from caregiving (*U* = 1965.5, *z* = −3.941; *p* ≤ 0.001); find support services aimed at the caregiver (*U* = 2491, *z* = −2.032; *p* = 0.042); share experiences with other caregivers online (*U* = 2438, *z* = −2.274; *p* = 0.023); and use internet interventions (*U* = 2444.5, *z* = −2.307; *p* = 0.021).

Concerning need variables, the i) number of hours spent caring, as well as caregivers perceived ii) physical and iii) mental health, were significantly associated with the frequency of internet use for several caregiving specific purposes. Participants spending more hours providing care report more frequent use of the internet to search for legal, fiscal, and financial information (*r*_s_ = 0.185, *p* = 0.022). Caregivers appraising their physical health differently (worse, similarly, better) diverge concerning their use of the internet to gather generic information on dementia [*H*(2) = 17.459, *p* ≤ 0.001], and to learn strategies to provide quality care [*H*(2) = 7.497, *p* = 0.024]. *Post-hoc* tests (Bonferroni-adjusted alpha level) show that only the difference between the caregivers assessing their physical health as similar and those appraising it as worse is statistically significant (*p* < 0.001, and *p* = 0.020, respectively). Caregivers appraising their physical health as worse report more frequent use of the internet for such purposes. Inversely, concerning using the internet to manage the negative psychological consequences of caregiving, the frequency is higher among caregivers appraising themselves as being in better physical health [*H*(2) = 7.797, *p* = 0.020]. Only the caregivers assessing their physical health as similar and those appraising it as better than their counterparts are significantly different (*p* = 0.048). The use of internet resources to gather information on dementia [*H*(2) = 6.888, *p* = 0.032], and to learn strategies to provide quality care [*H*(2) = 7, *p* = 0.044] differs according to mental health appraisal. *Post-hoc* tests show that such differences are only significant for the groups of caregivers assessing their mental health as similar in comparison with those appraising it as worse (*p* = 0.028, and *p* = 0.037), with a more frequent internet use among those perceiving their mental health more negatively.

### Factorial structure of questions on dementia and caregiving-related internet use

For the principal component analysis performed with the eight items assessing the frequency of internet use for dementia and caregiving-related internet resources, the KMO test value of 0.78 and the Bartlett's test of sphericity (χ^2^ = 1104.1, df = 28, *p* < 0.001) supported the factorability of the correlation matrix. The analysis yielded 1 factor with an eigenvalue >1.00, which explains 65% of the variance. The Parallel Analysis revealed only one component with an eigenvalue exceeding the corresponding criterion value for the randomly generated data matrix of the same size (500 replications). Hence, the advised number of dimensions from this analysis was 1. Table 4 presents the factor loadings for each item.

**Table 4:** Bias-corrected and accelerated (BCa) bootstrap 95% confidence intervals for loading values.

**Item description**	**Loadings**	**BCa confidence interval**
Gather information on disorders	0.815	0.711–0.870
Learn strategies to provide care	0.872	0.805–0.913
Find professional care services	0.857	0.794–0.902
Find support for carers	0.881	0.823–0.920
Manage psychological effects	0.857	0.781–0.903
Find legal/ financial information	0.717	0.564–0.801
Share experiences with carers	0.741	0.620–0.824
Use internet interventions	0.704	0.573–0.807

BCa, Bias-corrected and accelerated.

Assuming an unifactorial structure, an unweighted sum of all 8 items was computed to create a composite variable on the frequency of using dementia and caregiving-related internet resources (range = 8–32 points) and run the linear regression analysis. The average score for this sample was 20.8 points (SD 5.84).

### Frequency of using dementia and caregiving-related internet resources: Associated variables

Linear regression models (univariable and multivariable) to assess the impact of predisposing, enabling, and need variables on the frequency of using dementia and caregiving-related internet resources are displayed in Table 5. For univariable models, the frequency of using caregiving-related internet resources was significantly associated with the caregiver's age, relationship with the PwD, lifetime access to psychosocial interventions, caregiver's perceived physical health and, marginally, with caregiver' employment status, at a 5% significance level. The multiple regression considered eleven independent variables (see Table 5), including those with a *p* ≤ 0.25, or theoretically relevant (weekly hours spent caring; caregivers perceived mental health). The final model (backward method) retained the predisposing variable of caregivers' age; the enabling variables of caregivers' employment status, having a usual source of support to provide care, and lifetime access to psychosocial interventions; as well as the need variable of caregivers perceived physical health. All make a statistically significant unique contribution to the equation. Altogether, this model explained 19.4% of the variance (adjusted *R*^2^), *F*_(6, 136)_ = 6.681, *p* < 0.001.

**Table 5:** Univariable and multivariable linear regression models for the association between the frequency of using dementia and caregiving-related internet resources (composite) and predisposing, enabling, and need variables in informal dementia carers.

**Independent variables**	**Crude**				**Model (Adjusted) (*****N*** = **143)**			
	**B**	**95% CI**		***p***	**β**	**95% CI**		***p***
**Predisposing**								
IC age (years)	−0.085	−0.165	−0.005	0.039	−0.110	−0.192	−0.028	0.009
IC gender								
Female	2.772	−2.025	7.569	0.255	–	–	–
Male	Ref.							
Years of schooling	0.105	−0.112	0.322	0.340	–	–	–
Marital status								
Partnered	−0.823	−2.650	1.045	0.385	–	–	–
Non-partnered	Ref.							
Relationship with the person with dementia								
Offspring	2.978	0.344	5.612	0.027	–	–	–
Spouses	Ref.							
Cohabitation								
Yes	0.190	−1.920	2.299	0.859	–	–	–
No	Ref.							
CR age (years)	−0.062	−0.161	0.036	0.212	–	–	–
CR gender								
Female	0.687	−1.376	2.750	0.512	–	–	–
Male	Ref.							
**Enabling**								
IC employment status								
Employed	1.928	−0.013	3.869	0.051	2.558	0.540	4.576	0.013
Not employed	Ref.							
Support for caregiving								
Yes	−1.567	−3.520	0.386	0.115	−2.554	−4.538	−0.569	0.012
No	Ref.							
Lifetime access to psychosocial intervention								
Yes	2.447	0.610	4.285	0.009	2.731	0.950	4.512	0.003
No	Ref.							
**Need**								
Type of dementia								
Alzheimer's	1.517	−0.346	3.380	0.110	–	–	–
Vascular	0.122	−2.147	2.392	0.915	–	–	–
Other/unknown	Ref.							
CR dependence level								
Total/severe	0.402	−1.591	2.394	0.691	–	–	–
Mild/moderate	Ref.							
Hours caring (week)	0.005	−0.012	0.022	0.552	–	–	–
Caregiving duration (years)	0.027	−0.212	0.266	0.823	–	–	–
IC perceived physical health								
Much worse or worse	2.339	0.485	4.197	0.014	3.591	1.601	5.581	<0.001
Similar	Ref.							
Better or much better	0.846	−1.783	3.476	0.526	2.520	−0.348	5.388	0.085
Carer perceived mental health								
Much worse or worse	0.370	−1.544	2.284	0.703	–	–	–
Similar	Ref.							
Better or much better	0.939	−1.595	3.472	0.465	–	–	–
*R*^2^ (*R*^2^ adjusted)						0.228 (0.194)	

IC, informal caregiver; CR, care recipient; Ref., reference category; CI, confidence interval.

The composite variable for the frequency of using dementia and caregiving internet resources may range from 8 to 32 points.

For each 1-year increase in the caregivers' age, there is a decrease of 0.110 points, on average, in the frequency of using caregiving-related internet resources. Having usual support for caregiving is associated with a lower frequency of using caregiving-related internet resources (−2.554 points on average) than not having such support. Conversely, associated with a higher frequency of using caregiving-related internet resources is having used a psychosocial intervention at some point (2.731 points on average), being employed (2.558 points on average), and perceiving the own physical health as worse than the health of counterparts (as compared to appraising it as similar; 3.591 points on average). The variable making the strongest unique contribution to explain the frequency of dementia and caregiving-related internet use, when the variance explained by other variables in the model is controlled for, is the caregivers' perceived physical health as worse (Beta = 0.303; contribution to the total *R*^2^ of 7%).

## Discussion

This exploratory study sought to investigate online information and support-seeking behaviors of informal dementia caregivers who can use the internet autonomously. No similar study was carried out in Portugal and, internationally, the research topic is scantly researched. A disaggregated analysis of internet use for eight different caregiving-related purposes, together with a focus on the frequency of use rather than on the dichotomy of use/non-use are two distinctive elements of our study design compared to research produced to date. We have concluded for a moderate frequency of using dementia and caregiving-related internet resources. Across all the eight specific purposes investigated, a “frequent” internet use was never observed in more than 46% of the sample. The average score of the composite measure of dementia and caregiving-related internet use was 20.8 in 32 possible points. In an American study, Kim et al. have concluded that only 59.1% of dementia caregivers were health-related internet users (15); while in a South-European study, 47.5% of caregivers have received dementia services online (13). In our study, the caregivers using internet resources, regardless of the frequency, range from 49% (for using internet interventions) to 93% (to gather information on dementia). Hence, the prevalence of caregiving-related internet use is higher than the prevalence reported in previous studies, and higher than health-related internet use estimated for the general Portuguese population [49% (33)]. However, there is a high variability on the measurement of health, dementia, and/or caregiving-related internet use [e.g., a single item in (15) on internet resources “related with being a caregiver”; vs. asking about receiving dementia services online in (13)]. Therefore, no direct comparisons can be made. Moreover, internet usage trends are rapidly evolving, and distinct study timeframes may be influential. Our finding may also relate to the fact that we have studied a sample of frequent internet users.

In describing internet use for distinct purposes, we concluded that caregivers show less information and support-seeking behaviors for their own benefit than for directly benefiting the person in care. Caregivers may perceive the use of internet resources as an additional caregiving task rather than a help-seeking behavior (34). Moreover, reluctance in help-seeking for mental health is a well-known issue and may contracyclically be more expressed by the persons who are more in need (35). Using the internet to share experiences with other caregivers was infrequently reported in our sample, in line with findings from another European study (13).

This study also sought to identify variables associated with the frequency of using dementia and caregiving-related internet resources. First, we compared dementia caregivers who characterize differently on predisposing, enabling, and need variables on the frequency of using the internet for each of the eight dementia and caregiving-related purposes under study. We concluded that associations of predisposing, enabling, and need variables varied across specific caregiving-related internet use purposes (Supplementary Table). Second, we resorted to linear regression models to assess the impact of predisposing, enabling, and need variables on a composite measure of such usage frequency. The final multivariable linear regression model suggests that being younger, not having a usual source of support to provide care, having used a face-to-face psychosocial intervention at some point, being employed, and perceiving the own physical health as worse when compared to counterparts, are associated to a higher frequency of using dementia and caregiving-related internet resources. Perceived physical health and lifetime access to psychosocial interventions were the variables with the strongest unique contribution to explaining such frequency. These results highlight the relevance of including enabling and need variables in future research. The association of lifetime access to psychosocial interventions with caregiving-related internet use was a new addition from our study to the field.

Our findings confirmed the associations of age (5, 13, 15), relationship with the person in care (in the univariable model) (13, 15), and perceived health status (5), with caregiving-related internet use (however, in (16), there was no relationship between self-rated health and health-related internet use). We do not confirm the role of formal education (5, 13, 15), caregiving duration (13, 17), or number of hours spent caring (15), even though we found the last to be associated with the frequency of internet use to find legal/fiscal information (*r*_s_ = 0.185). Contrary to the conclusions of other studies (14, 15) and to what we have previously hypothesized, we did not find an association of using internet resources with the degree of dependence of the person in care. Studies concluding for such relationship showed either more (14) or less (15) use of health-related internet resources by caregivers of severely dependent persons.

While it was reported that persons facing barriers in accessing conventional services are more likely to use health-related internet resources (36), we conclude otherwise, with caregivers reporting lifetime use of psychosocial services reporting more frequent use of caregiving-related internet resources. Moreover, Kim et al. have concluded that emotional stress is associated with health-related internet use (15, 16). We used an indicator of perceived psychological health, and we could not confirm an association of this with the overall use of caregiving-related internet resources. However, more frequent use of the internet to find information on dementia, or to find strategies to provide quality care, was seen among caregivers who perceive themselves in worse mental health. In referring to research on conventional support services, the relationship of caregivers' mental health with service use is equivocal and can go both ways (higher or lower use for worse mental health) (37).

While this study is attentive to research gaps, its findings must be seen in the context of study limitations. Since this study follows a cross-sectional design, we are not able to determine causality. For instance, we could not determine whether caregivers who appraise their physical health more favorably are more likely to seek web resources to manage the psychological effects of caregiving or whether using such resources holds a positive effect on perceived physical health.

Another caveat concerns the use of a convenience sample. As participants were recruited by advertising through community projects and caregivers' associations, they may be more likely to show help and information-seeking behaviors. Also, by studying internet use and resorting to an e-survey, one must account for the influential role of education on internet use (38). Highly educated participants comprised most of our sample, which differs from the typical characterization of Portuguese informal caregivers as low educated (23). Moreover, most of our sample comprises children of the PwD (younger caregivers). As such, the frequency of internet use may be higher in this sample as if it was appraised in a sample better representing less-educated individuals and older spousal caregivers. As individuals who can use the internet tend to be more educated, have higher income, and be younger (39), study findings must not be applied to the mainstream population of caregivers but rather to a segment of similar profile to this sample.

To date, no predictive model was proposed to explain the frequency of using dementia and caregiving-related internet resources. Given the exploratory nature of this study, we cannot suggest such a model either. Our findings should be seen as hypotheses-generating, especially in informing on enabling and need variables associated with using caregiving-related internet resources.

Future work would benefit from more complex sampling methods, longitudinal design, and even more comprehensive measurements of internet use for caregiving-related purposes. An examination of additional factors is encouraged in considering the small to moderate associations found for most variables. Future research must also shed light on caregivers' eHealth literacy, i.e., on the ability to locate, analyze, and act upon internet-based health information. Evidence is missing on whether internet resources used by caregivers are understood and useful. Moreover, whether caregivers search for information online merely through search engines or use apps to help with finding information, is an open question.

This study has offered insights into which frequency and for which purposes informal dementia caregivers seek dementia and caregiving-related information and support through the internet. We concluded for a moderate frequency of using the web to reach such resources. The internet is primarily used to find general information about dementia and, to a much less extent, to find support for caregivers themselves through formal services or sharing experiences with peers. This research also identifies predisposing, enabling, and need variables associated with both the overall and the purpose-specific frequency of using dementia and caregiving-related internet resources. We offer insights on the role of under-researched enabling and need variables, which provide contextual information and may allow an in-depth understanding of online support-seeking behaviors. Our research shows that in addition to being younger, not having a usual source of caregiving support, having a history of using face-to-face psychosocial interventions, being employed, and perceiving the own physical health more negatively, are associated with a more frequent use of dementia and caregiving-related internet resources.

## Data availability statement

The raw data supporting the conclusions of this article will be made available by the authors, without undue reservation.

## Ethics statement

The studies involving human participants were reviewed and approved by the Ethics Committee for Health of the São João University Hospital Center/Faculty of Medicine of the University of Porto (reference 208/18). The patients/participants provided their written informed consent to participate in this study.

## Author contributions

ST: conceptualization, methodology, formal analysis, investigation, writing—original draft, funding acquisition, and responsible for ensuring that the descriptions are accurate and agreed by all authors. CP: conceptualization, methodology, writing—review and editing, and supervision. CC-S: methodology and formal analysis. AF: conceptualization, resources, writing—review and editing, and supervision. All authors contributed to the article and approved the submitted version.

## Funding

This publication was supported by National Funds through the Portuguese Foundation for Science and Technology, I.P. (FCT), in the scope of the project UIDP/04255/2020 [A publicação deste trabalho foi financiada por fundos nacionais através da FCT Fundação para a Ciência e a Tecnologia, I.P., no âmbito do projeto/centro de investigação “CINTESIS—Financiamento Programático; UIDP/04255/2020”]. ST was individually funded by the Portuguese Foundation for Science and Technology (FCT) (D/BD/135496/2018). The funding source was not involved in the research.

## Conflict of interest

The authors declare that the research was conducted in the absence of any commercial or financial relationships that could be construed as a potential conflict of interest.

## Publisher's note

All claims expressed in this article are solely those of the authors and do not necessarily represent those of their affiliated organizations, or those of the publisher, the editors and the reviewers. Any product that may be evaluated in this article, or claim that may be made by its manufacturer, is not guaranteed or endorsed by the publisher.

## Supplementary material

Supplementary Table Frequency of internet use per dementia and caregiving-related purpose, and associations with predisposing, enabling and need variables (*N* = 158).
