# Supplementary material for: Use of dementia and caregiving-related internet resources by informal caregivers: A cross-sectional study
Source: Front Med (Lausanne). 2022 Sep 14;9:978635. doi: 10.3389/fmed.2022.978635 (PMC9518752; doi:10.3389/fmed.2022.978635)
Supplement: Supplementary Table — Frequency of internet use per dementia and caregiving-related purpose, and associations with predisposing, enabling and need variables (N = 158). [file Table_1.pdf]

Supplementary table 1. Frequency of internet use per dementia and caregiving-related purpose, and associations with predisposing, enabling and need variables (N=158).

	Use of dementia and caregiving-related internet resources																							
	Gather information on disorders			Learn to provide care			Find professional care services			Manage psychological effects			Find support for caregiver			Find legal/ financial information			Share experiences with caregiver			Use internet interventions		
	Mdn (IQR)	Mean Rank/$r_s^{\#}$	***p***	Mdn (IQR)	Mean Rank/r_s	***p***	Mdn (IQR)	Mean Rank/r_s	***p***	Mdn (IQR)	Mean Rank/r_s	***p***	Mdn (IQR)	Mean Rank/r_s	***p***	Mdn (IQR)	Mean Rank/r_s	***p***	Mdn (IQR)	Mean Rank/r_s	***p***	Mdn (IQR)	Mean Rank/r_s	***p***
**Predisposing variables-caregiver**																								
Age (years) [a]	3 (1)	-.073	.364	3 (1)	-.167	**.037**	3 (2)	-.127	.113	3 (1.5)	.017	.830	3 (1)	-.068	.398	2.5 (1)	-.170	**.034**	2 (2)	-.025	.757	1 (1)	.010	.902
Gender [b]			.882			.903			.929			.644			.550			.118			.759			.054
Female	3 (1)	79.39		3 (1)	77.91		3 (2)	79.07		3 (1)	79.38		3 (1.5)	79.45		3 (1)	79.68		2 (2)	78.77		2 (1)	80.39	
Male	3,5 (1)	81.63		3 (1)	79.86		3 (2)	77.69		3 (2)	72		2 (2.5)	69.36		2 (2)	53.29		2 (2)	83.86		1 (0)	49.21	
Years of schooling [a]	3 (1)	-.018	.825	3 (1)	.017	.834	3 (2)	.080	.319	3 (1.5)	.019	.813	3 (1)	-.043	.597	2.5 (1)	.010	.906	2 (2)	-.030	.709	1 (1)	.060	.459
Relationship with the PwD [b]			**.018**			**.014**			.283			.771			.484			.093			.389			.528
Offspring*	3 (1)	79.33		3 (1)	77.91		3 (2)	77.03		3 (1)	75.07		3 (1)	76.51		3 (1)	77.46		2 (2)	76.73		2 (1)	76.38	
Spouses	3 (3)	58.40		3 (3)	55.98		3 (3)	67.04		3 (3)	77.85		3 (2)	69.91		3 (2)	61.54		1 (2)	68.72		1 (2)	70.65	
Marital status [b]			.937			.416			.593			.556			.928			.205			.202			.538
Partnered	3 (1)	79,74		3 (1)	75.46		3 (2)	77.29		3 (1)	80.93		3 (1)	79.29		2 (2)	74.34		2 (2)	74.93		1 (1)	77.09	
Non-partnered	3 (1)	79.22		3 (1)	80.85		3 (2)	80.97		3 (2)	76.78		2 (2)	78.66		3 (1.5)	83.23		2 (2)	83.68		1 (2)	81.20	
Cohabitation [b]			.525			.755			.448			.884			.397			.279			.822			.724
Yes	3 (1)	76.14		3 (1)	75.35		3 (2)	78.69		3 (2)	77.33		3 (3)	78.91		3 (3)	78.93		2 (2)	76.50		1 (2)	77.76	
No	3 (1)	80.70		3 (1)	77.59		3 (1)	73.08		3 (1)	76.23		3 (1)	72.55		2 (1)	70.73		2 (2)	78.16		2 (1)	75.23	
**Predisposing variables-care recipient**																								
Age (years) [a]	3 (1)	-.068	.395	3 (1)	-.070	.388	3 (2)	-.086	.285	3 (1.5)	-.151	.058	3 (1)	-.043	.591	2.5 (1)	-.022	.788	2 (2)	.070	.383	1 (1)	.019	.810
Gender [b]			.153			.956			.956			.516			.623			.252			.833			.615
Female	3 (1)	82.50		3 (1)	80.99		3 (2)	79.12		3 (1)	77.58		3 (1)	77.92		3 (1)	81.05		2 (2)	79.46		2 (1)	80.06	
Male	3 (1)	71.98		3 (1)	70.47		3 (2)	78.69		3 (2)	82.66		3 (3)	81.70		3 (3)	72.20		2 (2)	77.87		1 (2)	76.36	

Abbreviations: n= number of participants; PwD= person with dementia; Mdn= Median; IQR= interquartile range; CR= care recipient; values in bold highlight the statistically significate associations.

[a] Tested by Spearman's Rho Test

[b] Tested by Mann-Whitney U Test

[c] Tested by Kruskal Wallis H Test

*Categories excluded for group comparison due to the small number of participants in the category: the relationship ‘other’.

[#] Mean rank is presented for Mann-Whitney U Test and Kruskal Wallis H Test; *r* values are presented for Spearman's Rho Test.

Supplementary table 1. Frequency of internet use per dementia and caregiving-related purpose, and associations with predisposing, enabling and need variables (N=158). (continuation)

	**Use of dementia and caregiving-related internet resources**																							
	Gather information on disorders			Learn to provide care			Find professional care services			Manage psychological effects			Find support for caregiver			Find legal/ financial information			Share experiences with caregiver			Use internet interventions		
	Mdn (IQR)	Mean Rank/r_s[#]	***p***	Mdn (IQR)	Mean Rank/r_s	***p***	Mdn (IQR)	Mean Rank/r_s	***p***	Mdn (IQR)	Mean Rank/r_s	***p***	Mdn (IQR)	Mean Rank/r_s	***p***	Mdn (IQR)	Mean Rank/r_s	***p***	Mdn (IQR)	Mean Rank/r_s	***p***	Mdn (IQR)	Mean Rank/r_s	***p***
**Enabling variables**																								
Caregiver employment status [b]			.151			**.034**			**.003**			.543			.138			.568			.635			.603
Employed	3 (1)	81.55		3 (1)	81.75		3 (1)	85.01		3 (2)	79.08		3 (1)	81.37		2 (1)	78.51		2 (2)	76.29		2 (1)	78.80	
Not employed	3 (1.75)	71.72		3 (2)	67.25		3 (2)	63.98		3 (2)	74.66		3 (2)	70.73		2 (2)	74.39		2 (2)	79.63		1 (1.75)	75.23	
Support for caregiving [b]			.847			.784			.609			.068			.134			.063			**.031**			.066
Yes	3 (1)	78.44		3 (1)	75.83		3 (2)	76.22		3 (1)	72.83		2 (1)	73.65		2 (2)	72.17		1 (2)	72.06		1 (1)	72.98	
No	3 (1)	77.15		3 (1)	77.71		3 (2)	79.87		3 (2)	86.14		3 (2)	84.43		3 (2)	85.61		2 (2)	87.30		2 (2)	85.63	
Psychosocial interventions(access)[b]			.138			.295			.120			**<.001**			**.042**			.394			**.023**			**.021**
Yes	4 (1)	84		3 (1)	81		3 (1)	83.89		3 (2)	92.47		3 (2)	85.65		3 (1)	80.99		2 (2)	86.34		2 (2)	86.25	
No	3 (1)	74.19		3 (1)	74.09		3 (2)	73.25		3 (2)	64.88		2 (1)	71.53		2 (2)	75.04		1 (2)	70.86		1 (1)	70.94	
**Need variables**																								
Type of dementia [c]			.799			.167			.435			.465			.342			.873			.995			.998
Alzheimer's disease	3.5 (1)	70.31		3 (1)	73.75		3 (1.25)	72.89		3 (2)	68.87		3 (2)	73.28		3 (1)	70.76		2 (2)	69.77		2 (1)	69.33	
Vascular dementia	3.5 (1)	72.16		3 (1)	60.55		3 (1)	65.26		3 (1)	65.18		3 (2)	66.15		2 (1)	66.65		2 (2)	69.21		1 (2)	69.74	
Other/unknown	3 (1)	65.70		3 (2)	60.55		3 (1)	63.50		2 (1)	77.93		2.5 (2.75)	61.15		2 (3)	69.28		1.5 (1.75)	69		1.5 (1)	69.76	
CR dependence level [b]			.172			.490			.874			.957			.914			.125			.407			.725
Mild/moderate	3 (1)	72.67		3 (1)	74.31		3 (1)	77.75		3 (1)	78.76		3 (1.5)	79.03		2 (2)	70.46		2 (1)	74.50		2 (1)	80.15	
Total/severe	4 (1)	82.23		3 (1)	79.13		3 (2)	78.88		3 (2)	78.36		3 (1)	78.24		3 (2)	81.81		2 (2)	80.50		1 (1)	77.67	
Caregiving duration (years) [a]	3 (1)	.064	.426	3 (1)	.028	.7267	3 (2)	.016	.847	3 (1.5)	.085	.293	3 (1)	.074	.358	2.5 (1)	.102	.210	2 (2)	-.114	.157	1 (1)	-.007	.928
Hours caring (week) [a]	3 (1)	-.021	.790	3 (1)	-.008	.922	3 (2)	.001	.992	3 (1.5)	.082	.305	3 (1)	.015	.850	2.5 (1)	.185	**.022**	2 (2)	.103	.200	1 (1)	-.062	.443
Caregiver physical health [c]			**<.001**			**.024**			.213			**.020**			.223			.230			.064			.179
Much worse or worse	4 (1)	91.66		3.5 (1)	85.92		3 (1.75)	82.86		3 (2.75)	83.02		3 (2)	82.25		3 (2)	82.94		2 (2)	84.16		2 (2)	82.66	
Similar	3 (1)	61.42		3 (1)	66.34		3 (1)	69.95		3 (1)	65.39		2 (1)	69.93		2 (2)	69.81		1 (1)	67.29		1 (1)	69.72	
Better or much better	3 (1)	75.42		3 (1)	73.94		3 (1)	81.06		3 (2)	91.00		3 (1)	83.09		2 (2)	77.33		2 (2)	83.00		2 (2)	82.20	
Caregiver mental health [c]			**.032**			**.044**			.582			.567			.690			.833			.739			.638
Much worse or worse	4 (1)	84.47		3 (1)	83.55		3 (2)	80.36		3 (2)	77.93		3 (2)	79.27		3 (1.5)	77.99		2 (2)	77.27		1 (1)	77.92	
Similar	3 (1)	63.72		3 (2)	63.76		3 (2)	72.24		3 (1)	74.58		2 (1)	73.74		2 (1)	79.27		2 (2)	77.53		2 (2)	83.28	
Better or much better	3 (1)	81.77		3 (1)	76.63		3(0.25)	81.08		3 (2)	86.23		3 (1)	82.88		3 (2)	82.06		2(2.5)	84.56		1 (1)	73.40	

Abbreviations: n= number of participants; PwD= person with dementia; Mdn= Median; IQR= interquartile range; CR= care recipient; values in bold highlight the statistically significate associations.

[a] Tested by Spearman's Rho Test, [b] Tested by Mann-Whitney U Test, [c] Tested by Kruskal Wallis H Test

*Categories excluded for group comparison due to the small number of participants in the category: the relationship ‘other’.

[#] Mean rank is presented for Mann-Whitney U Test and Kruskal Wallis H Test; *r* values are presented for Spearman's Rho Test.
